# Diabetic Foot Ulcer as an Independent Risk Factor for Cardiovascular Mortality: A Systematic Review and Meta-Analysis With Qualitative Synthesis of Cardiac Biomarker Evidence

**DOI:** 10.7759/cureus.106672

**Published:** 2026-04-08

**Authors:** Muhammad Farhan, Tirath Patel, Sheikh Bilal Ahmed Shahbaz Ahmed, Arwa Ossama Abdelrazek, Rafi Aliefiyanto, Muhammad Yahya Khattak, Hawraa Al Shabout, Fikri Aminullah, Naufal Rasendriya Akhmad, Ahmed Taleb Ibrahim, Mohammad Ayman Abdulraziq, Carolyn K Edmondson, Samantha Olivia Colucci, Adekunle E Omole

**Affiliations:** 1 Department of Medicine, Ajman University, Ajman, ARE; 2 School of Medicine, Trinity Medical Sciences University, Kingstown, VCT; 3 Faculty of Medicine, Universitas Airlangga, Surabaya, IDN; 4 Clinical Medicine, American University of Antigua, St. John, ATG; 5 Student Affairs, American University of Antigua, St. John, ATG; 6 School of Engineering Medicine, Texas A&M University, Houston, USA

**Keywords:** b-type natriuretic peptide (bnp), cardiac biomarker, cardiovascular-related mortality, diabetic foot ulcer (dfu), diastolic dysfunction, prolonged qtc interval

## Abstract

Diabetic foot ulcer (DFU) is closely correlated with cardiovascular mortality, but the quantitative synthesis that underlies this association is limited. Additionally, the literature is limited in its reporting of the role of cardiac biomarkers and their influence on cardiovascular mortality among patients with DFU. This study aimed to examine the strength of association between DFU and cardiovascular disease (CVD)-specific mortality, and the role of specific cardiac parameters in increasing CVD-specific mortality risk. This systematic review and meta-analysis were conducted in compliance with the Preferred Reporting Items for Systematic Reviews and Meta-Analyses 2020 guidelines. First, a narrative synthesis summarized hazard ratios (HRs) of markers such as B-type natriuretic peptide (BNP) levels, left ventricular ejection fraction, and diastolic dysfunction. For the quantitative meta-analysis, pooled HRs with 95% confidence intervals (CIs) of included studies were used as the primary measure of association between CVD mortality and DFU. Heterogeneity was quantified using the I² statistic (I² ≥ 50% indicating substantial heterogeneity). Subgroup analyses were performed for glycemic control (HbA1c ≥7%) and ulcer severity; continuous meta-regression was performed for mean age and median follow-up time (standardized to months). All analyses were conducted at the study level using aggregated data. A sensitivity analysis was also performed, including prospective cohort and case-control studies, and excluding studies at high risk of bias. Publication bias was assessed visually using funnel plots and by employing Egger’s regression test. All statistical analyses were performed using software JAMOVI version 2.4.11. In total, 14 studies were included in the final meta-analysis, of which three were included in the qualitative synthesis. The pooled HR of 2.57 (k = 14, 95% CI = 1.85, 3.28, p < 0.001, I² = 98.45%) indicates a significant association between DFU and CVD mortality. The 95% prediction interval was 0.14-47.88, reflecting the wide range of true effects expected across heterogeneous DFU study settings. Sensitivity analysis stratifying by ulcer severity (k = 4) and study design (k = 8) reduced heterogeneity to I² of 0%; however, this result should be interpreted cautiously given the small number of studies per stratum (k = 4), which limits statistical power to detect true heterogeneity and likely reflects over-stratification rather than true homogeneity. However, the qualitative synthesis found that BNP (>100 pg/mL) was significantly associated with CVD mortality (HR = 4.846, 95% CI = 2.482, 9.461, p < 0.001). Similarly, QTc prolongation was associated with CVD-specific mortality (HR = 5.465, 95% CI = 2.818, 8.112, p = 0.039). This meta-analysis and qualitative synthesis highlight that cardiac biomarkers, including BNP (>100 pg/mL) and QTc prolongation, showed associations with CVD mortality in narrative synthesis; however, these findings are hypothesis-generating only, derived from three studies, and require confirmation in larger prospective studies before clinical recommendations can be made.

## Introduction and background

Diabetic foot ulcer (DFU) is a serious microvascular complication of diabetes mellitus, and is becoming an indicator of extensive systemic disease, not a regional foot pathology [1]. The prevalence of DFU is up to 25% of patients with long-term disease, which constitutes a significant share of diabetes-related morbidity, mortality, and healthcare costs in the world [2]. There is growing evidence that persons with DFU have significantly higher cardiovascular disease (CVD) events and cardiovascular deaths than patients with diabetes without ulcers. This correlation remains despite the modulation of classic cardiovascular risk factors and indicates that DFU is a manifestation of extensive endothelial dysfunction, inflammation, autonomic neuropathy, and increased atherosclerosis [3]. Despite this substantial clinical burden, the pathophysiological processes linking DFU to adverse cardiovascular outcomes have not been comprehensively analyzed, and there is a lack of integrated evidence on the interactions among cardiac biomarkers, cardiac functional indices, and DFU, as well as on long-term mortality. Such a knowledge gap limits risk clustering and prevents prompt interventions in a population already at disproportionately high risk [4].

Cardiac biomarkers such as B-type natriuretic peptide (BNP), left ventricular ejection fraction (LVEF), and indices of diastolic dysfunction have been proposed as part of the mechanisms underlying the excess cardiovascular risk in patients with DFU [5]. BNP and its inactive precursor, N-terminal pro-BNP (NT-proBNP), are established biomarkers of myocardial strain and subclinical heart failure [6], while LVEF and diastolic dysfunction parameters reflect hemodynamic and structural cardiac changes driven by diabetes and ischemic cardiomyopathy [7,8]. Despite their established prognostic value in general cardiac populations, their predictive role specifically in DFU patients remains poorly characterized [9].

Patients with DFU develop a distinctive combination of systemic pathophysiological events that accelerate cardiovascular deterioration [10]. Chronic hyperglycemia, oxidative stress, persistent inflammation, autonomic neuropathy, and recurrent infectious insults collectively impair cardiac function through myocardial fibrosis, ventricular stiffness, and hemodynamic instability [11,12]. In this context, elevated BNP, reduced ejection fraction, and worsening diastolic dysfunction may serve not only as indicators of underlying cardiovascular impairment but also as precursors of systemic deterioration in the DFU population [13].

Several studies describe an increased level of BNP, inactive systolic impairment, and common diastolic dysfunction in subjects with DFU, which supports the hypothesis that occult cardiac dysfunction is associated with poor outcomes [14]. Nevertheless, DFU is significantly associated with cardiovascular mortality, even in the absence of measurement or statistical adjustment for these variables; the relationship between cardiac biomarkers and DFU is not entirely mediated [15]. This implies that there are two risks: in the presence of abnormal cardiac markers, DFU is a significant predictor of increased cardiovascular mortality, and in the presence of DFU, the risk further increases [16]. The question of the roles of cardiac biomarkers as mediators, modifiers, or parallel predictors thus requires an understanding that would enhance cardiovascular risk stratification and targeted surveillance of patients with DFU.

Despite this evidence, no comprehensive synthesis has quantified the strength of the DFU-CVD mortality association while simultaneously evaluating the prognostic role of cardiac biomarkers. This systematic review and meta-analysis aimed to quantify the association between DFU and cardiovascular mortality and to qualitatively assess whether cardiac biomarkers (BNP, LVEF, diastolic dysfunction) and ECG parameters further modify this risk.

## Review

Methodology

Protocol and Reporting Standards

This systematic review followed the Preferred Reporting Items for Systematic Reviews and Meta-Analyses (PRISMA) 2020 statement [17]. The protocol was prospectively registered with preselected eligibility criteria, search terms, and appraisal strategies, and was registered with PROSPERO (registration number: CRD420251267949). The research question was derived from the Population-Exposure-Outcome (PEO) framework presented in Table 1.

**Table 1:** Search string. The search string shows the specific combination of keywords, symbols, and Boolean operators (AND, OR, NOT) entered into the search engine or database to locate relevant information.

Database	Search string	Studies
PubMed	((“diabetic foot”[MeSH] OR “foot ulcer”[MeSH] OR (diabetic AND foot AND (ulcer OR wound)))) AND ((“natriuretic peptide, brain”[MeSH] OR “stroke volume”[MeSH] OR “diastole”[MeSH]) OR (Glycemic Control OR Ulcer Severity OR BNP OR “ejection fraction” OR “diastolic dysfunction”)) AND ((“mortality”[MeSH] OR “cardiovascular diseases”[MeSH]) OR (“cardiovascular mortality” OR death OR survival))	11
Scopus	((diabetic foot)) AND ((Glycemic Control OR Ulcer Severity OR BNP OR “ejection fraction” OR diastolic)) AND ((“cardiovascular mortality” OR “cardiac death”))	51
Cochrane	“Diabetic foot”[MeSH] AND (“natriuretic peptide, brain”[MeSH] OR “stroke volume”[MeSH] OR “diastole”[MeSH]) OR (Glycemic Control OR Ulcer Severity OR BNP OR Natriuretic peptide OR diastole dysfunction) AND “mortality”[MeSH]	67

Eligibility and Selection Criteria

Inclusion and exclusion criteria were determined utilizing the PEO framework [18]: Population (P): adult patients (≥18 years) with a diagnosis of DFU, irrespective of ulcer severity grade; Exposure (E): presence of DFU, with or without associated cardiac abnormalities (elevated BNP levels, reduced LVEF, and/or diastolic dysfunction); Outcome (O): long-term (≥1 year) cardiovascular mortality, glycemic control (HbA1C), and ulcer severity.

Inclusion criteria: Peer-reviewed publications written in English and published between January 1, 2000, and November 30, 2025. Observational studies (prospective or retrospective cohort, case-control) reporting on the association of DFU with cardiovascular mortality. If studies reported cardiac biomarkers such as BNP/NT-proBNP, LVEF, or diastolic dysfunction, they would be considered to establish the specific marker’s role in increasing long-term CVD mortality in a defined DFU population. The study population must consist of adult patients (≥18 years) with a clinical diagnosis of DFU, irrespective of Wagner or University of Texas classification grade. Studies must report extractable quantitative data on the association (e.g., hazard ratios (HRs)) or provide sufficient data for their calculation.

Exclusion criteria: Studies not specifically conducted in a DFU cohort (e.g., general diabetes, heart failure, or post-myocardial infarction populations without separate DFU analysis). Case reports, case series, editorials, letters, conference abstracts, narrative reviews, and animal studies. Studies where the primary outcome was not CVD-specific mortality. Studies published in languages other than English.

Search Strategy

A systematic literature search was conducted to identify studies investigating the association between DFU and CVD mortality, as well as cardiac function parameters. Electronic databases, including PubMed, the Cochrane Library, and Scopus, were searched from January 1, 2000, to November 30, 2025. Search strategies combined controlled-vocabulary terms (e.g., MeSH headings) with free-text keywords listed in Table 1 [19]. No limits were imposed at the initial level except for date and language filters. To ensure thoroughness, the reference lists of included studies and previous reviews were manually searched to identify additional cardiac biomarker-related associations, but there was limited information on specific markers. All retrieved citations were imported into EndNote X9, and duplicates were removed.

Research Question

Among adults with diabetes mellitus, is the presence of DFU associated with increased cardiovascular mortality? Additionally, do cardiac functional parameters (BNP, LVEF, and diastolic dysfunction) further modify this association?

Study Selection, Data Extraction, and Data Items

The study’s selection process was conducted in accordance with the PRISMA 2020 guidelines. Initial screening included independent reviewers reading the articles’ titles and abstracts. Subsequently, the independent reviewers conducted a full-text review of the articles. Regarding the reviewers' disagreement, a consensus was reached through the involvement of another reviewer.

A standardized, pilot-tested data extraction form was developed before full review, and 14 eligible studies were included. The extracted data encompassed the following domains: study identification (authors, publication year); methodology (study design, total sample size (N)); participant details (population characteristics such as age, gender, HbA1c, ulcer severity); and study parameters (follow-up duration in years). Crucially, data on the exposure variables of interest were extracted, including BNP levels, LVEF, and the presence/severity of diastolic dysfunction. Concurrently, details on outcome variables were recorded, with a primary focus on the cardiovascular mortality rate. To assess potential confounding, data on glycemic control (e.g., HbA1c ≥7%) and ulcer severity were collected. Data were independently extracted using a pre-piloted electronic form and cross-verified for accuracy. Any disagreements were resolved through discussion or by consulting another reviewer.

Methodological Quality Assessment and Data Synthesis

The quality assessment was conducted using the Risk of Bias in Non-randomized Studies of Interventions (ROBINS-I) Cochrane tool to determine the risk of bias among included prospective, retrospective cohort, and case-control studies. The evidence was categorized as low, moderate, serious, and critical risk of bias (ROB) [20]. The Robvis software was used to generate the ROB plots for visual illustration [21].

The data synthesis was performed in two stages: narrative and quantitative synthesis. Narrative synthesis was performed for predictors such as BNP levels, LVEF, and diastolic dysfunction, given limited reporting in only three studies each. However, a quantitative (meta-analytic) synthesis was performed for DFU- and CVD-specific mortality using a random-effects model with the DerSimonian-Laird estimator. A 95% prediction interval was calculated alongside the pooled HR to convey the expected range of true effects across settings.

The narrative synthesis summarized the characteristics, methodological quality, and direction of association for each study (BNP levels, LVEF, and diastolic dysfunction). For the quantitative meta-analysis, the primary measure of association was the pooled HR with 95% confidence interval (CI) from included studies. Heterogeneity was quantified using the I² statistic (I² ≥50% indicating substantial heterogeneity). Subgroup analyses were performed for glycemic control (HbA1c >7%) and ulcer severity by pooling study HRs within each stratum. Continuous meta-regression was performed for mean age and median follow-up time, with all follow-up durations standardized to months before analysis (range = 14-120 months). All analyses were conducted at the study level using aggregated data and are therefore subject to ecological bias; no individual-level causal inference should be drawn from these analyses. A sensitivity analysis was also performed, including prospective cohort and case-control studies, and excluding studies at high risk of bias. Publication bias was assessed visually using funnel plots and Egger’s regression test. All statistical analyses were performed using software JAMOVI version 2.4.11.

Results

**Figure 1:**
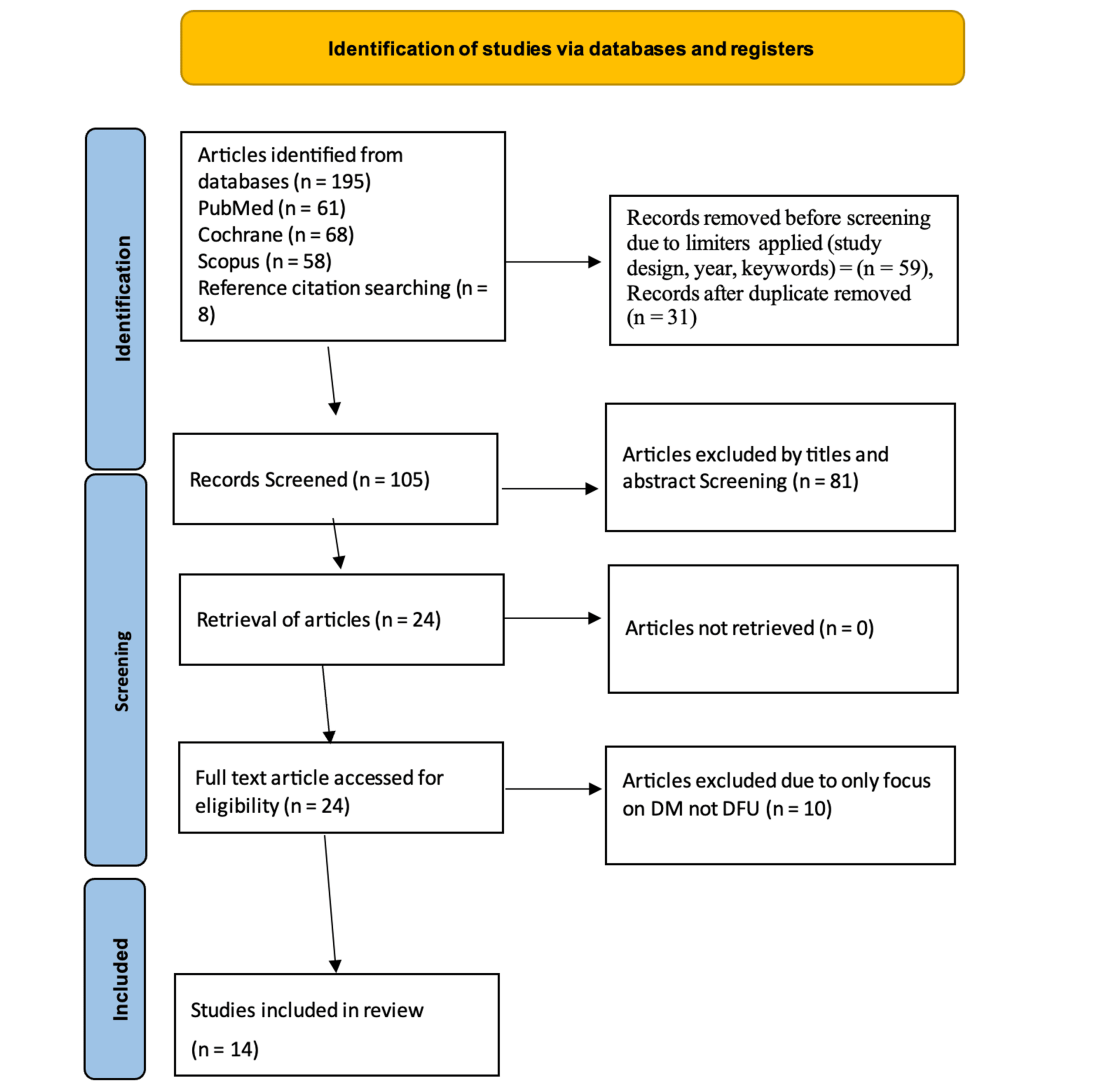
PRISMA flow diagram for systematic review. This diagram illustrates the systematic process of identifying, screening, assessing for eligibility, and including studies for the review. PRISMA: Preferred Reporting Items for Systematic Reviews and Meta-Analyses

The systematic search process and study selection are shown in the PRISMA 2020 flow diagram in Figure 1.

The literature search yielded 195 articles. After applying limiters/filters, 59 articles were excluded. Moreover, 31 duplicates were removed through EndNote 27. Overall, 81 out of 105 articles were excluded after screening of titles and abstracts. The remaining 24 articles underwent full-text eligibility assessment. Of the 24 articles, 10 were excluded because they included non-DFU patients, and the remaining 14 were included in the synthesis. All 14 articles were included in the quantitative analysis because they reported CVD mortality among patients with DFU. However, only three articles that reported BNP, LEVF, and diastolic dysfunction were included in the qualitative synthesis. The characteristics of the included studies are presented in Table 2.

**Table 2:** Characteristics of included studies. This table provides a comprehensive summary of the included studies, detailing the author, year, country, sample size, condition, key patient demographics (male percentage, mean/median age), follow-up duration, HbA1c levels, ulcer severity, biomarkers (BNP, LVEF, and diastolic dysfunction), CVD mortality analysis, and five-year survival rate. DFU: diabetic foot ulcer; T2D: type 2 diabetes; IQR: interquartile range; NR: not reported; BNP: B-type natriuretic peptide; LVEF: left ventricular ejection fraction; CVD: cardiovascular disease

Authors	Country	Design	Condition	N	Age	Gender (% male)	Follow-up duration	HbA1c (HbA1c > 7%)	Ulcer Severity	BNP	LVEF	Diastolic dysfunction	CVD mortality (HR at 95% CI) from univariate analysis	Survival rate at 5 years
Rubio et al., 2020 [22]	Spain	Retrospective observational cohort study	DFU	338	71 years (62–80)	65.40%	Median: 96 months (IQR: 74.4–114 months)	1.71 (1.27–2.29)	1.19 (1.08–1.32)	NR	NR	NR	2.10 (1.58–2.78)	60%
Rubio et al., 2017 [23]	Spain	Retrospective observational cohort study	DFU	345	71 (32–95)	65.80%	Median: 33.6 months (IQR: 15.6–61.2 months)	0.96 (0.83–1.01)	NR	NR	NR	NR	2.15 (1.83–3.88)	60%
Forde et al., 2019 [24]	Ireland	Retrospective observational cohort study	DFU	98	57.0 ± 11.7	68.70%	60 months	1.27 (0.39–4.14)	NR	NR	NR	NR	2.36 (0.25–3.29)	86.70%
Adeleye et al., 2020 [25]	Nigeria	Prospective observational cohort	DFU	323	57.2 ± 11.4	54.20%	NS	0.502 (0.110–2.291)	7.558 (2.285–24.999)	NR	NR	NR	2.161 (0.66–7.067)	NR
Jeyaraman et al., 2019 [26]	Australia	Retrospective observational cohort study	DFU	513	55.9 ± 12.3	62.80%	Median: 69.6 months (IQR: 37.2–117.6 months)	1.24 (0.98, 1.5)	NR	NR	NR	NR	1.78 (1.34, 2.36)	75.40%
Brownrigg et al., 2014 [27]	UK	Retrospective clinic-based cohort study	DFU vs. DM	869	68.8 ± 13.3	69.50%	Median: 43.2 months (IQR: 39.6–50.4 months)	1.02 (1.01–1.03)	NR	NR	NR	NR	2.53 (0.98–6.49)	55%
Iversen et al., 2009 [28]	Norway	Population-based prospective cohort study (HUNT 2)	DFU vs. DM only vs normal	65,126	67.2 ± 14.0	56.80%	120 months	1.11 (1.06–1.16)	NR	NR	NR	NR	2.56 (2.15–3.04)	50% (males) to 55% (females) at 5 years
Pinto et al., 2008 [29]		Prospective cohort study (5-year follow-up)	DM + DFU vs. DM only	225 (102 DM + DFU, 122 DM only)	DM + DFU: 66.7 ± 9.8 years DM only: 66.9 ± 13 years	DM + DFU: 55.8, DM only: 54.4	60 months	2.43 (1.20–4.92)	NR	NR	NR	LVH: 1.48 (0.99–2.19)	8.67 (4.28–17.56)	NR
Faglia et al., 2013 [30]	Italy	Retrospective cohort study	DFU	278	69.5 + 10.9	70.30%	Mean: 43.2 ± 2.4 months	0.778 (0.664–0.911)	1.024 (0.465–2.254)	61/71 (85.9%) who died had BNP >100 pg /mL. HR: 4.846 (2.482.9.461): p < 0.001	NR	NR	1.923 (1.26–2.957)	50.90%
Novida et al., 2022 [31]	Indonesia	Case-control study	DFU deceased vs. DFU survived	358	Cases: 57.71 ± 9.83. Controls: 55.84 ± 9.80	NR	NS	NR	6.80 (3.77–12.27)	NR	NR	NR	2.93 (1.64–5.25)	NR
Wang et al., 2018 [32]	China	Prospective cohort study	DFU	331	70 (31 to 96)	61.3293051	Median 48 months	NR	NR	NR	NR	NR	2.011 (1.106–3.657)	NR
Lan et al., 2025 [33]	Australia	Prospective cohort study	DFU	513	64.8 ± 12.3	76.30%	13–15 months	NR	NR	NR	NR	NR	1.81 (0.68, 4.86)	NR
Vitale et al., 2024 [34]	Italy	Prospective observational cohort study	DFU vs. DM controls	895 DFU vs. 15,773 (T2D) controls	70.1 ± 9.6	67.40%	Median 89.0 months	1.06 (1.03–1.08)	NR	NR	NR	NR	1.502 (1.346–1.676)	-
Qiu et al., 2022 [35]	China	Retrospective case-control study	DFU vs. DM controls	88	67 ± 12	65.70%	Median 74.4 months	NR	NR	NR	NR	NR	3.277 (1.392–7.715)	NR

Reporting of Risk of Bias

**Figure 2:**
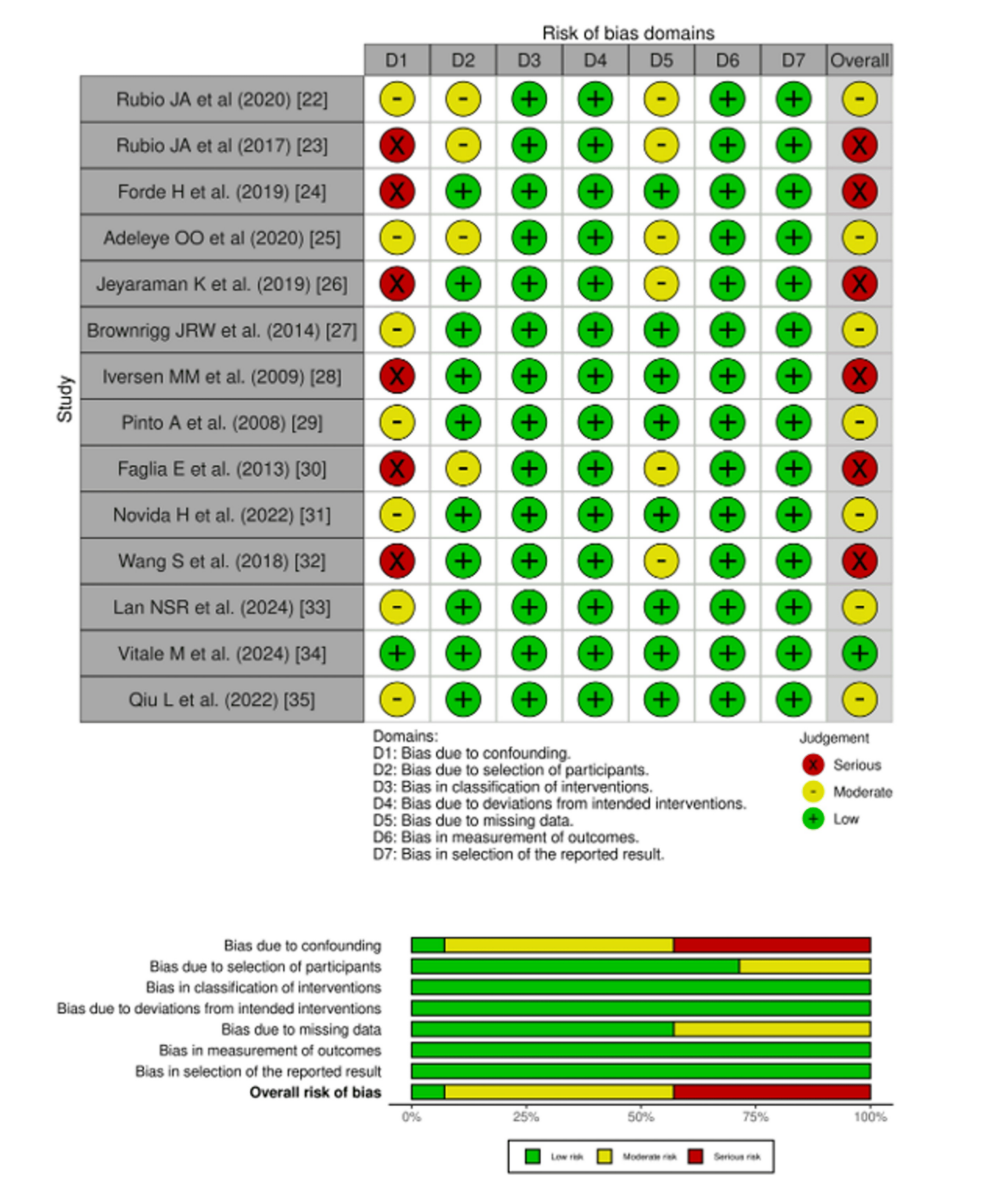
Risk of bias assessment summary from the included studies. This diagram presents the specific risk of bias judgments for each of the 14 included studies across all seven methodological domains, using the ROBINS-I tool. References: [22-35]. ROBINS-I: Risk of Bias in Non-randomized Studies of Interventions

The majority of studies (seven) exhibited moderate ROB [22,25,27,29,31,33,35], and only one study exhibited low ROB [34]. However, six studies were downgraded to high ROB due to bias in the D1 domain (confounding bias) [23,24,26,28,30,32]. Overall, the evidence was moderate to low ROB (Figure 2).

Meta-Analysis

**Figure 3:**
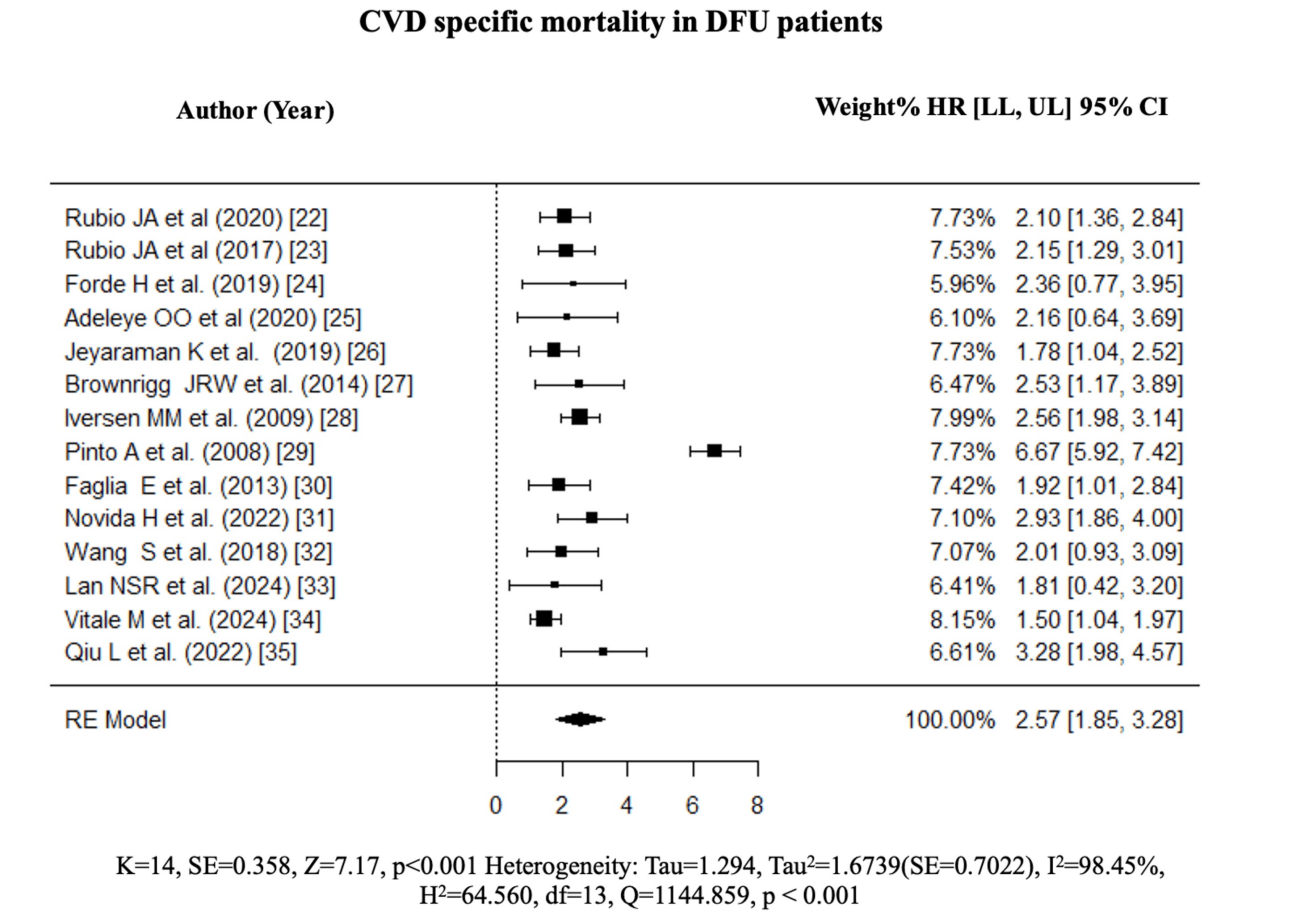
Forest plot of the pooled hazard ratio for CVD-specific mortality among DFU patients. k = 14; HR = 2.57, 95% CI = 1.85–3.28; 95% prediction interval = 0.14–47.88; I² = 98.45%. References: [22-35]. CVD: cardiovascular disease; DFU: diabetic foot ulcer; HR = hazard ratio; CI: confidence interval; LL: lower limit; UL: upper limit

The pooled HR ratio was 2.57 (k = 14, 1.85, 3.28, p < 0.001), indicating a significant association between DFU and CVD mortality [22-35] (Figure 3).

**Figure 4:**
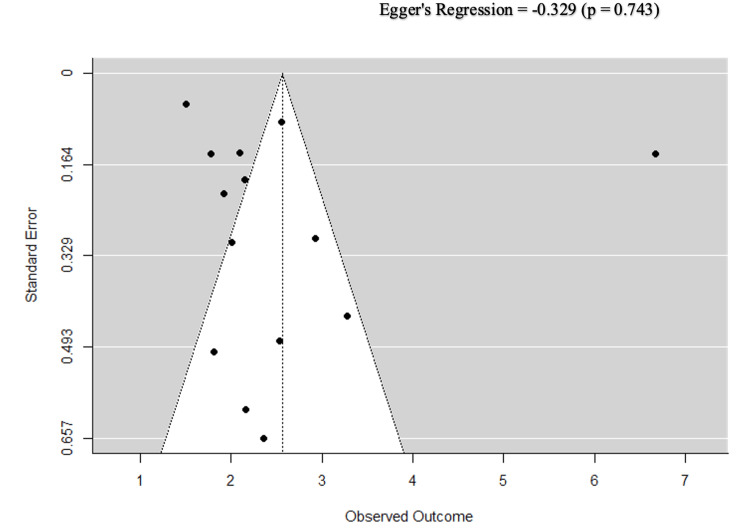
A funnel plot to check the existence of publication bias.

The 95% prediction interval was 0.14-47.88, reflecting the range of true effects across different study settings. The high heterogeneity (I² = 98.45%, p < 0.001) is attributable to genuine clinical variability, including differences in CVD subtypes, ulcer severity grades, and study designs, confirmed by the sensitivity analysis excluding high-ROB studies, which preserved high heterogeneity (I² = 97.54%). The symmetrical funnel plot and Egger’s regression test (p = 0.743) confirmed the absence of publication bias (Figure 4).

Moderator Analysis

**Figure 5:**
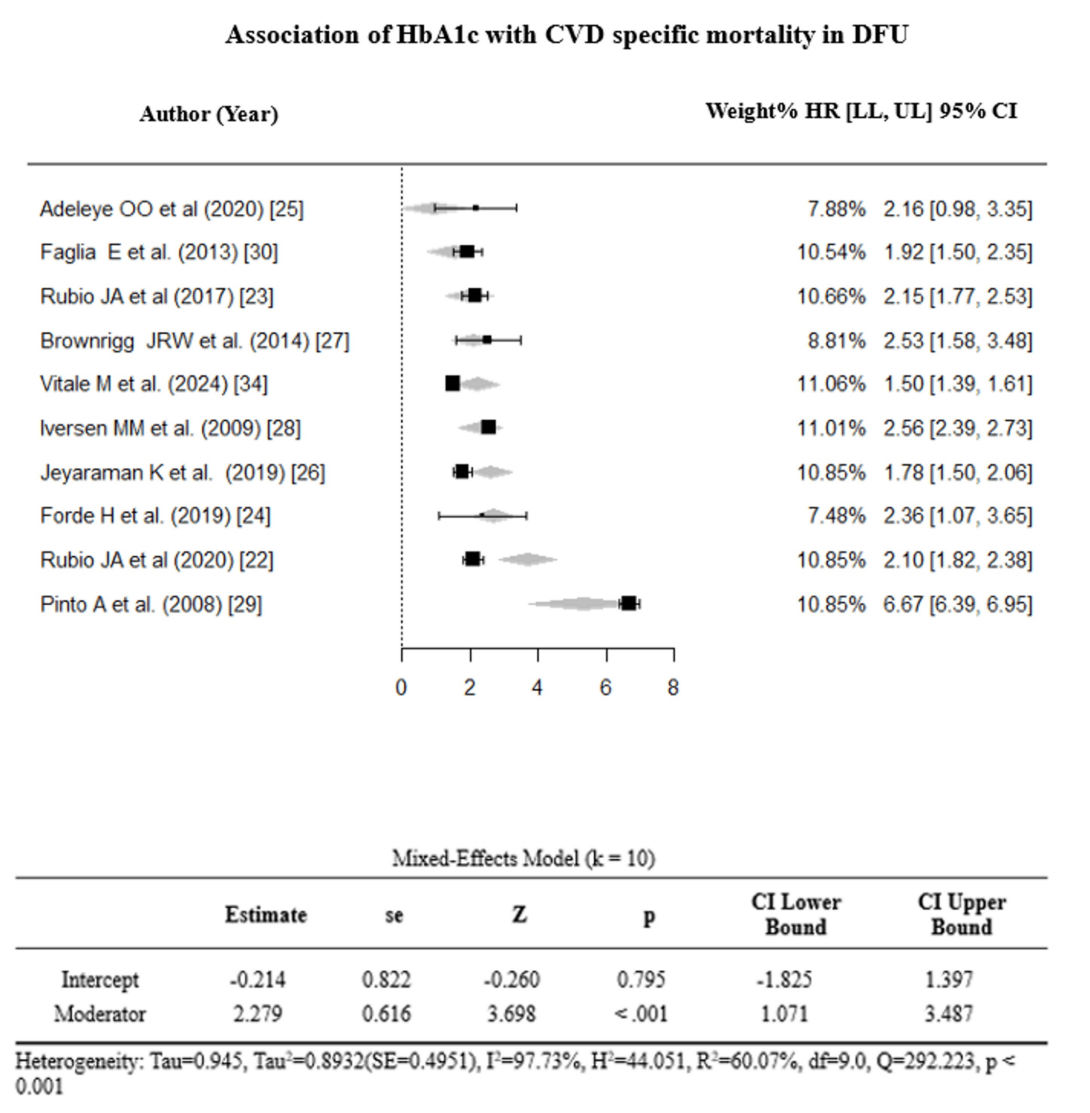
A forest plot to illustrate moderator analysis for HbA1c. References: [22-30,34]. CVD: cardiovascular disease; DFU: diabetic foot ulcer; HR = hazard ratio; CI: confidence interval; LL: lower limit; UL: upper limit

**Figure 6:**
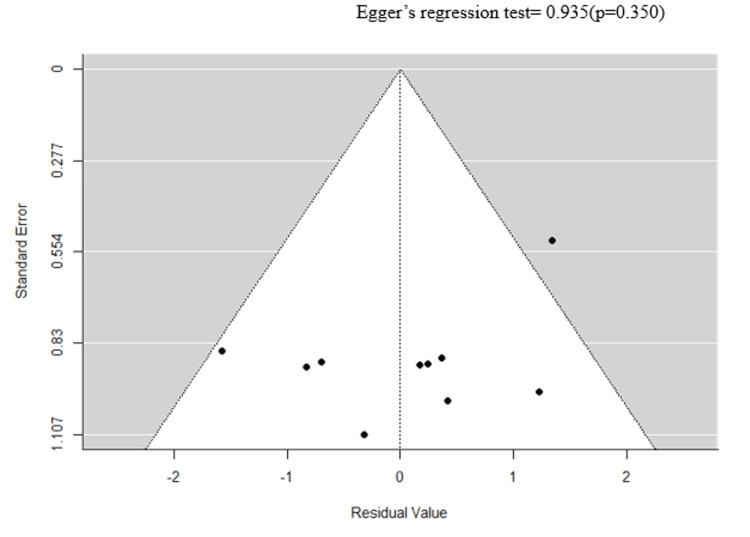
A funnel plot illustrating moderator analysis for HbA1c.

Subgroup analyses and continuous meta-regression were performed for the following four study-level variables: HbA1c >7%, ulcer severity, mean age, and median follow-up time. Continuous meta-regression for mean age and median follow-up time (standardized to months; range = 14-120 months) was not statistically significant (follow-up: β = −0.00007, 95% CI: −0.0077 to 0.0076, p = 0.985) (see Appendices). Subgroup analysis indicated that studies with a higher proportion of patients with HbA1c >7% were associated with significantly greater CVD mortality risk (k = 10, HR = 2.279, 95% CI = 1.071-3.487, p < 0.001, I² = 97.73%). These findings are based on study-level aggregate data and are subject to ecological bias; no individual-level causal inference can be drawn. Egger’s regression test was non-significant (p > 0.05), confirming the absence of publication bias (Figures 5, 6).

Sensitivity Analysis

Sensitivity analyses were performed by stratifying by ulcer severity and study design and by excluding the six high-ROB studies. The summary of the sensitivity analysis is presented in Table 3.

**Table 3:** Summary of the sensitivity analysis. *: I² = 0% with k = 4 studies per stratum. This likely reflects insufficient Q-test power (over-stratification) rather than true homogeneity and should not be interpreted as absence of heterogeneity. ROB: risk of bias; HR: hazard ratio; CI: confidence interval

Analysis	k	Pooled HR	95%Cl	I²	Egger’s p
Primary analysis (all studies)	14	2.57	1.86–3.27	98.45%	0.743
Excluding high-ROB studies	8	2.57	1.75–3.78	97.54%	0.152
Ulcer severity stratum	4	2.15	1.74–2.67	0.00%*	0.369
Prospective/Case-control study design only	8	2.58	1.79–3.71	86.01%	0.549

**Figure 7:**
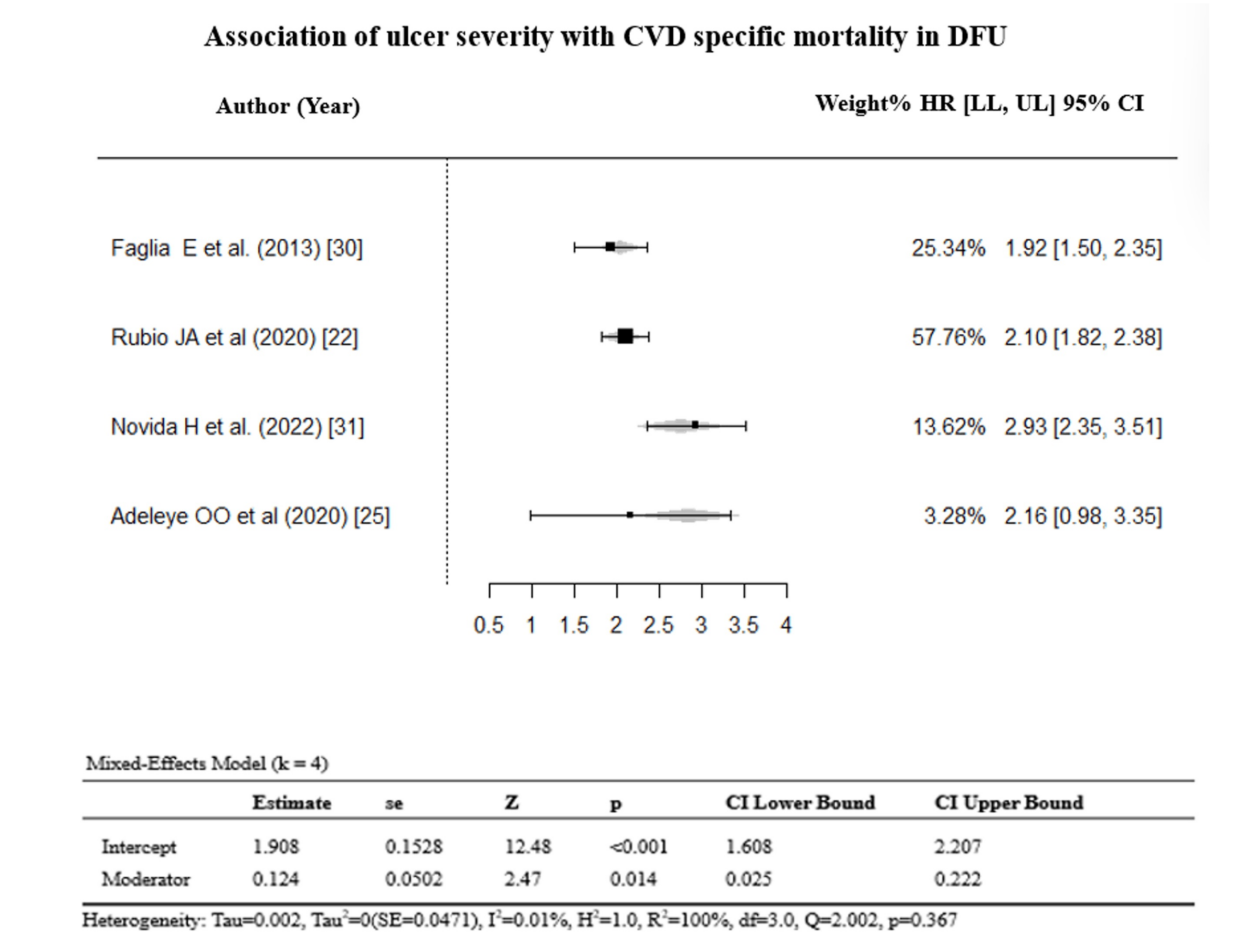
Sensitivity analysis of studies reporting ulcer severity. References: [22,25,30,31]. CVD: cardiovascular disease; DFU: diabetic foot ulcer; HR = hazard ratio; CI: confidence interval; LL: lower limit; UL: upper limit

**Figure 8:**
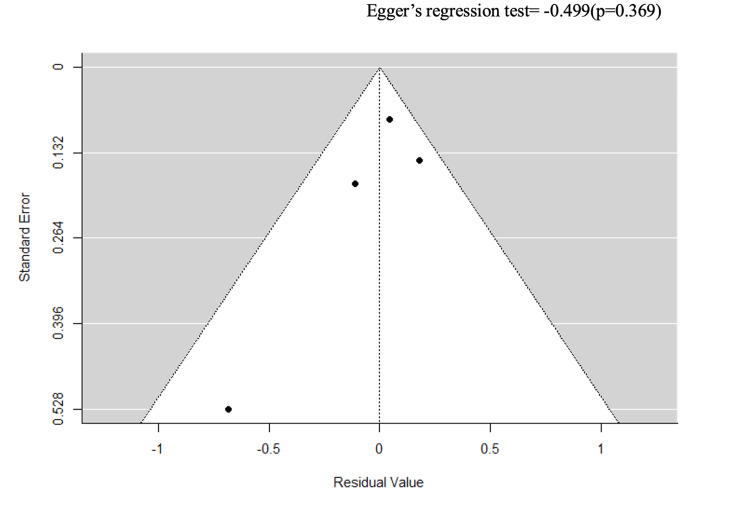
A funnel plot for sensitivity analysis on ulcer severity.

**Figure 9:**
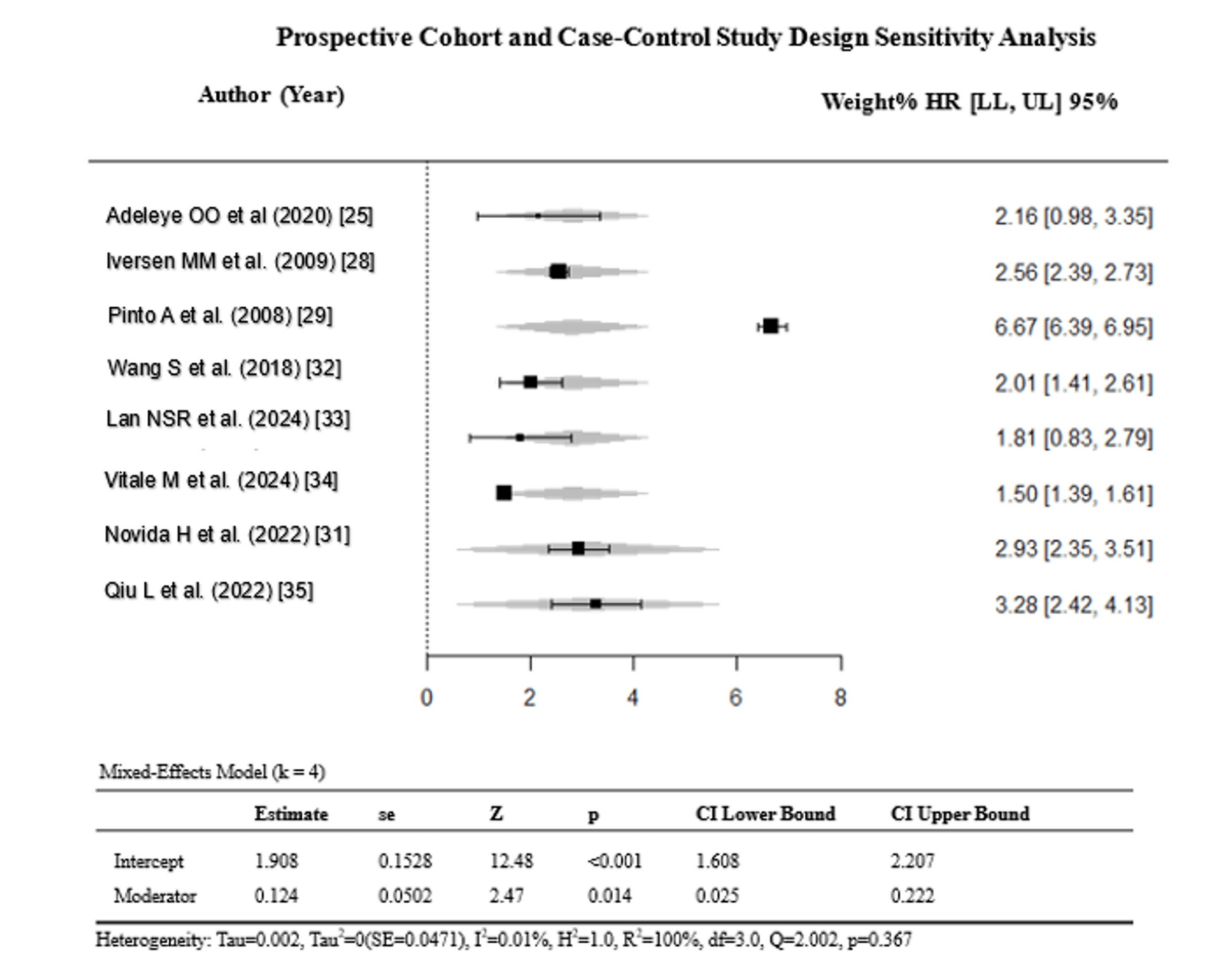
Sensitivity analysis excluding retrospective cohort studies. References: [25,28,29,31-35]. CVD: cardiovascular disease; DFU: diabetic foot ulcer; HR = hazard ratio; CI: confidence interval; LL: lower limit; UL: upper limit

**Figure 10:**
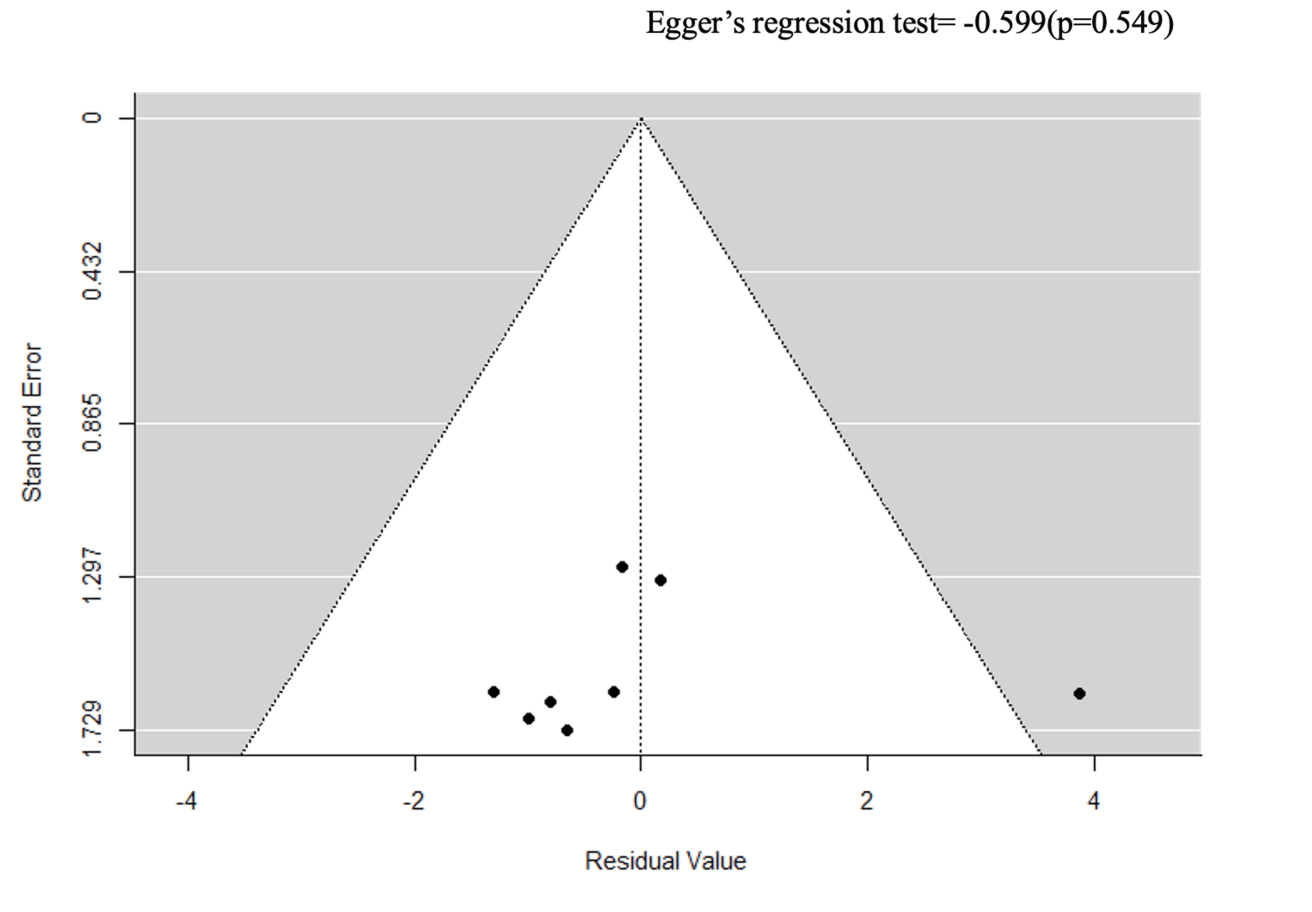
A funnel plot for sensitivity analysis (excluding retrospective cohort studies).

Stratification by ulcer severity (k = 4) and study design (k = 8) reduced heterogeneity to an I² of 0% (Egger’s p = 0.369 and p = 0.549) (Figures 7-10).

**Figure 11:**
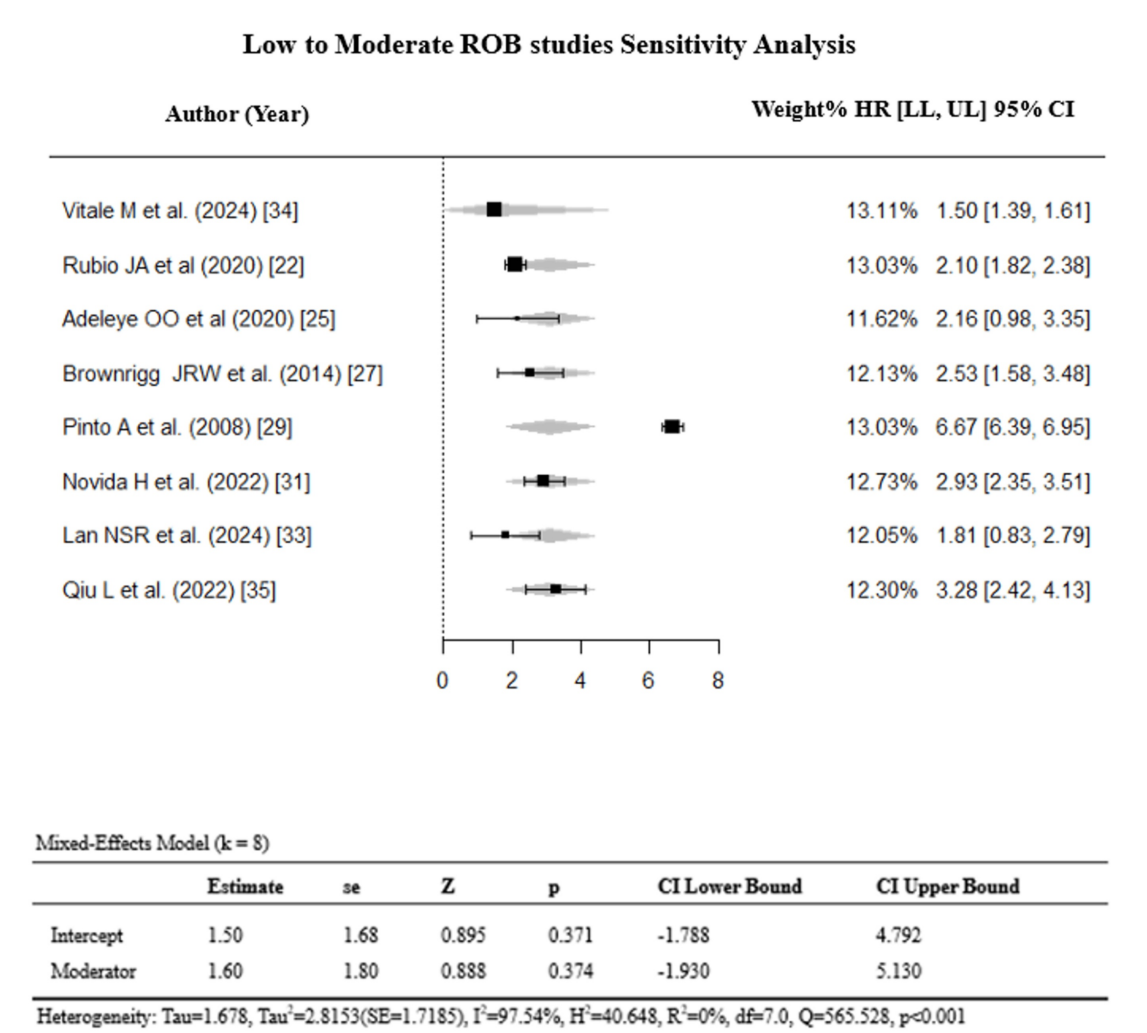
Sensitivity analysis (excluding high ROB studies). References: [22,25,27,29,31,33-35]. ROB: risk of bias; HR = hazard ratio; CI: confidence interval; LL: lower limit; UL: upper limit

**Figure 12:**
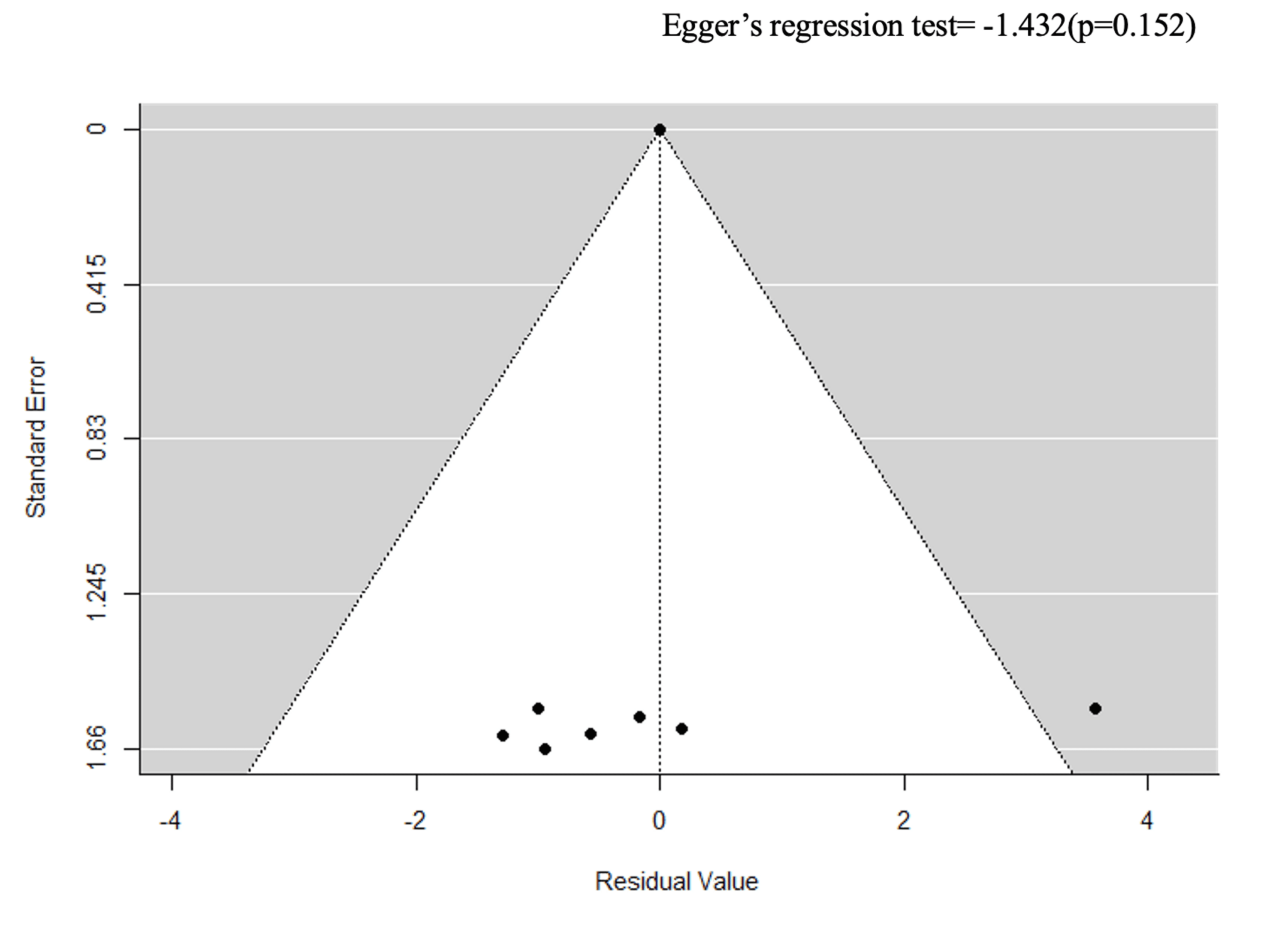
A funnel plot for sensitivity analysis (excluding high ROB studies). ROB: risk of bias

However, this apparent reduction must be interpreted with caution: each stratum contained only k = 4 studies, substantially limiting the statistical power of the Q-test, and an I² of 0% under these conditions likely reflects over-stratification rather than true homogeneity. Critically, excluding high-ROB studies did not reduce heterogeneity (I² = 97.54%), confirming that the primary source of variability is genuine clinical heterogeneity, i.e., differences in CVD subtypes, ulcer severity classification systems, and study designs, rather than methodological bias (Figures 11, 12).

The overall direction of effect (HR ≈2.5) was preserved after high-ROB exclusion, supporting robustness of the main finding.

Qualitative Synthesis

The findings from three studies on cardiac biomarkers collectively support the conclusion that DFU is an independent predictor of CVD morbidity and mortality [29,30,32]. There is also a complex association among cardiac biomarkers, which serve as important mediators. The condition is marked by a high rate of subclinical cardiac pathology (myocardial injury, stress, autonomic dysfunction), which can be well-measured using increased cardiac biomarkers. These biomarkers are likely to increase the risk of CVD mortality [29,30,32]. However, the current literature has shown limited focus on these three cardiac biomarkers, which may explain the mediation effect observed in quantitative analyses. Due to limited reporting across the included studies, three of the 14 studies that focused on cardiac biomarkers were narratively synthesized [29,30,32].

Role of Cardiac Wall-Related Findings Via ECG in Predicting Cardiovascular Disease Mortality

Pinto et al. (2008) [29] conducted a prospective cohort study with a mean follow-up of five years to compare patients with DM plus DFU with those with DM only and to assess the impact of cardiac ECG markers on CVD-related mortality. The authors found that the cardiovascular risk profile and the rate of new cardiovascular events in DFU were worse and more frequent after five years. DFU presence was the most predictive independent variable for cardiovascular morbidity and mortality in a multivariate model (presence of DFU versus age and HbA1c), with an HR of 8.67 (95% CI = 4.28-17.56). This makes DFU a clinically relevant factor. Concerning the prevalence of subclinical CVD, patients with DFU were more likely to have major ECG abnormalities, an intima-media thickness >0.9, a carotid plaque, left ventricular hypertrophy, and abnormal regional wall motion compared with patients without DFU. At the five-year follow-up, patients with DFU had a higher incidence of new-onset cardiovascular events compared with diabetic patients without limb lesions. Statistically significant differences were observed in the incidence of new cases of diabetic retinopathy, but no difference in the incidence of new renal failure diagnoses. During the five-year follow-up, 14 (13.7%) deaths were recorded in patients with diabetic foot versus 10 (8.1%) (p < 0.05) in patients without diabetic foot, and most of these deaths in both groups were attributable to cardiovascular causes [29].

Role of B-type Natriuretic Peptide Levels in Predicting Cardiovascular Disease Mortality

Faglia et al. (2013) [30] also conducted a retrospective cohort study to assess the association between DFU and all-cause mortality. The authors reported a high HR of 6.04 (95% CI = 2.38-15.33) among DFU patients. In multivariate analysis, 61/71 (85.9%) DFU patients who died had BNP >100 pg/mL, and reported an HR of 4.846 (2.482-9.461, p < 0.001). It suggests that BNP levels greater than 100 pg/mL might be associated with the cause of death among DFU patients [30]. Although the evidence is based on retrospective studies, the findings appear substantial and need further exploration for validation.

Role of QTc Prolongation in Predicting Cardiovascular Disease Mortality

Wang et al. (2018) [32] also conducted a prospective cohort study in China to examine whether QTc interval prolongation may be an independent risk factor for DFU outcomes. The study enrolled 331 hospitalized Chinese tertiary patients with type 2 diabetes and DFU. The QTc interval was determined from the baseline ECG using Bazett’s formula. The QTc interval was used to divide participants into two groups: prolonged (QTc ≥440 ms) and normal (QTc <440 ms). These patients were followed for an average of 48 months to assess ulcer healing, recurrence, nonfatal cerebral or cardiovascular events, cerebral cardiovascular death, cardiac death, and all-cause mortality. Cox proportional hazards models examined outcomes of QTc interval prolongation and a 1-SD increase in QTc interval. In univariate Cox proportional-hazards models, QTc interval prolongation was associated with higher all-cause mortality (HR = 1.621, 95% CI = 1.040-2.526, p = 0.013) and cardiac mortality (HR = 2.011, 95% CI = 1.106-3.657, p = 0.019). In multivariate analysis, QTc prolongation was an independent risk factor for cardiac mortality (HR = 5.465, 95% CI = 2.818-8.112, p = 0.039). The QTc interval was linked with cardiac mortality (HR = 6.883, 95% CI = 4.153-9.613, p = 0.012). It should be noted that Wang et al. applied a QTc threshold of 440 ms using Bazett’s correction formula. This threshold does not align with current American College of Cardiology (ACC)/American Heart Association (AHA) sex-specific definitions (≥450 ms in males; ≥470 ms in females), and Bazett’s formula is known to overcorrect at elevated heart rates, which are common in DFU patients with autonomic neuropathy. These methodological limitations should be considered when interpreting this finding. Both univariate and multivariate analyses found that QTc prolongation may predict cardiac mortality in patients with DFU, but not ulcer healing or recurrence [32].

Discussion

Summary of Key Findings

The evidence was synthesized quantitatively and qualitatively. The finding that DFU more than doubles the risk of CVD-specific mortality, independent of classic cardiovascular risk factors, reinforces the concept that DFU represents a marker of advanced systemic vascular disease rather than an isolated local complication. Furthermore, poor glycemic control (HbA1c >7%) emerged as a significant study-level moderator, underscoring the importance of optimal glycemic management in mitigating cardiovascular risk in this population [22-30,34]. The heterogeneity was reduced; patients underwent a sensitivity analysis considering ulcer severity grade and a prospective study design, resulting in a substantial reduction in variability.

The extreme heterogeneity observed (I² = 98.45%) warrants careful interpretation of the pooled HR. The 95% prediction interval of 0.14-47.88 indicates the range within which the true effect is expected in a new, similar study setting. This wide interval reflects genuine clinical diversity across included studies, namely, differences in CVD subtypes, ulcer severity classification, follow-up duration, and patient demographics. Clinicians should interpret the pooled HR as indicative of the overall direction and approximate magnitude of association, rather than a precise effect size applicable uniformly across all DFU settings.

However, the qualitative synthesis also provides insightful findings and explains that biomarkers related to the heart, such as BNP (>100 pg/mL), were significantly correlated with the risk of mortality, which suggests that it may be used to increase the hazard of CVD outcomes in patients with DFU [30]. Similarly, QTc prolongation was also associated with cardiac mortality, further affirming the importance of ECG screening for patients at risk of cardiac-specific mortality [32]. Cardiac biomarkers, including BNP >100 pg/mL and QTc prolongation, demonstrated associations with CVD mortality in the narrative synthesis; however, these findings are derived from only three studies (Pinto et al. 2008 [29], Faglia et al. 2013 [30], Wang et al. 2018 [32]) with heterogeneous designs and limited sample sizes. Quantitative pooling of biomarker data was not feasible. These findings should be considered hypothesis-generating, and dedicated prospective studies with standardized biomarker protocols in DFU populations are required before these markers can be recommended for clinical risk stratification. Hence, these findings are crucial and provide potential implication for the clinicians and researchers to focus more on these specific cardiac markers to screen and monitor patients to mitigate the additional risk [29,30,32] However, the exact pooling of evidence was not possible due to limited reporting of these markers in the current literature, which emphasize the researchers to focus more on them to validate the findings.

Comparison of Findings With Existing Literature

The meta-analysis found a significant association between DFU and CVD mortality, with a pooled HR of 2.57 (k = 14, 1.85, 3.28, p < 0.001) [22-35]. This finding is also similar to the systematic review and meta-analysis conducted by Chen et al. (2023), who also found fatal MI (2.22, 95% CI = 1.09, 4.53) and fatal stroke (1.41, 95% CI = 0.61, 3.24) [36]. Hence, the findings are aligned to indicate that DFU patients are at risk of CVD-specific mortality.

Moreover, biomarkers, such as BNP levels (>100 pg/mL), were associated with a significant increase in mortality risk (HR = 4.846, 95% CI = 2.482, 9.461, p < 0.001). These findings are substantial, but the literature is limited in its exploration of the direct association of the BNP marker. However, some cross-sectional studies provide only a temporal association rather than a causal one. For instance, Demirtas et al. (2019) conducted a cross-sectional study and observed statistically significantly higher BNP levels (>100 pg/mL) among DFU patients (p < 0.05) compared with non-DFU patients [37]. However, in a prospective cohort study of diabetic patients, Julián et al. (2020) found a significant difference in NT-proBNP levels between type 2 diabetes (1,825 ng/L; interquartile range (IQR) = 808-4,363) and non-type 2 diabetes (1,530 ng/L; IQR = 611-3,344; p = 0.01) [38]. Hence, these findings are clinically relevant. Although the literature comparison of findings such as ECG-related QTc prolongation, LEVF, and diastolic dysfunction is promising, further longitudinal studies are required to pool HRs and provide more robust evidence to generalize the findings [32]. Hence, the need for additional prospective longitudinal studies of DFU patients to further validate the findings. The role of these markers is still in its early stages, but their potential relevance in clinical settings warrants consideration.

Clinical Implications

The evidence highlighted that glycemic control is poor (HbA1c >7.0), which makes patients with DFU have a greater risk of CVD mortality [22-35]. Clinicians should prioritize glycemic control to minimize cardiovascular risk. Early screening for BNP levels, QTc prolongation, and cardiac wall dysfunction is necessary to reduce the burden of high risk among DFU patients [29,30,32]. Therefore, ECG screening is necessary to identify high-risk individuals and improve patient outcomes in the early stages of the disease.

Strengths and Limitations

This systematic review and meta-analysis highlighted potential strengths, including being among the first studies to assess the role of these specific cardiac biomarkers and to synthesize evidence on the likelihood of increased mortality among DFU patients. The review synthesized evidence from primary studies, such as prospective cohort and case-control studies, reporting HRs to pool the evidence. The evidence is synthesized using standardized tools such as ROBINS-I and PRISMA 2020 guidelines. Publication bias was also assessed using the funnel plot and Egger’s regression test.

However, this study has some limitations. The pooled evidence showed substantial heterogeneity due to variability in CVDs (ischemic heart attack, cardiac failure, etc.), ulcer severity grading, and study designs. The current literature is limited to reporting cardiac-specific biomarkers, such as BNP levels, NT-proBNP, QTc interval prolongation, LEVF, and left ventricular hypertrophy, which limited our ability to pool evidence to determine the association of these markers with the likelihood of CVD-specific mortality among DFU [29,30,32]. Additionally, all subgroup and meta-regression analyses were conducted at the study level using aggregated data. These are therefore subject to ecological bias, and no individual-level causal inference can be drawn from any moderator or subgroup finding, including the HbA1c >7% association. The apparent reduction in heterogeneity to an I² of 0% in sensitivity analyses stratified by ulcer severity and study design should not be interpreted as true homogeneity. Each stratum contained only k = 4 studies, severely limiting the Q-test’s statistical power. This finding likely reflects over-stratification. Cardiac biomarker findings (BNP, QTc prolongation) are based on only three studies, precluding quantitative pooling. The exploratory nature of the narrative synthesis limits the generalizability of conclusions about biomarkers. The QTc threshold of 440 ms applied by Wang et al. does not reflect current ACC/AHA sex-specific thresholds (≥450 ms males; ≥470 ms females). Bazett’s correction formula is known to overcorrect at high heart rates common in DFU patients with autonomic neuropathy. Lastly, six of the 14 included studies were rated as high ROB under ROBINS-I, primarily due to inadequate confounding control [23,24,26,28,30,32]. Although sensitivity analysis excluding these studies preserved the direction of the main effect (HR ≈2.57), heterogeneity remained high (I² = 97.54%), indicating that variability is driven by clinical factors rather than bias alone. The pooled HR should nonetheless be interpreted with caution.

Future Recommendations

Future recommendations include investigating the associations among glycemic management, cardiovascular risk factors, and DFU outcomes, as well as conducting more longitudinal and prospective cohort studies. These studies may provide stronger evidence of the long-term effects of glycemic management on CVD mortality. Standardized cardiovascular screening, biomarker testing, and cardiac ECG are routine components of care for DFU patients. The investigation could examine optimal BNP and QTc cutoffs and their predictive value for cardiovascular mortality in this population. Interventions to improve cardiac health should include patients with DFU, particularly those with poor glycemic control or elevated cardiac biomarkers. As sodium-glucose co-transporter 2 inhibitors have been shown to lower blood glucose and cardiovascular risk, future studies should focus on developing therapeutic interventions that can control both. Future studies should standardize definitions of DFU severity, study methods, and CVD to reduce variation across the literature. This will improve comparability of studies and the quality of evidence to inform clinical practice. Subclinical cardiac pathology, such as myocardial damage and autonomic dysfunction, may affect CVD mortality rates in DFU patients; more research is needed. Further cardiac biomarker studies may help develop early intervention.

## Conclusions

This meta-analysis and qualitative synthesis highlight that although DFU is an independent risk factor for CVD-specific mortality, glycemic control (HbA1c ≥7) and ulcer severity play a valuable role in the moderation of CVD mortality in DFU patients. Moreover, cardiac biomarkers and ECG findings, such as BNP levels (>100 pg/mL) and QTc prolongation, provide useful prognostic value for CVD mortality. Clinically, these results highlight the need to closely monitor metabolic and cardiac parameters in this high-risk cohort of DFU, along with standard measurement of DFU ulcer severity. A standardized study design should be employed to gain further insight into variability in outcomes. The study identified a substantial association between these cardiac markers and CVD-specific mortality.

## Figures and Tables

**Figure 13:**
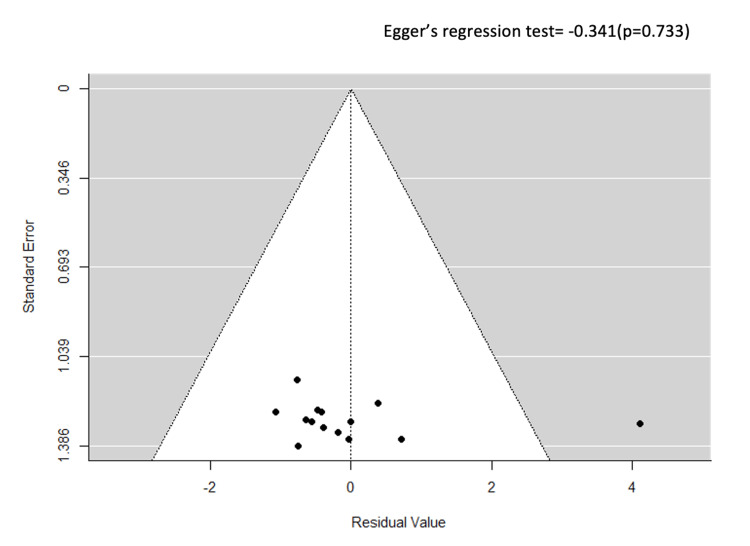
A funnel plot to illustrate moderator analysis for median follow-up time.

**Figure 14:**
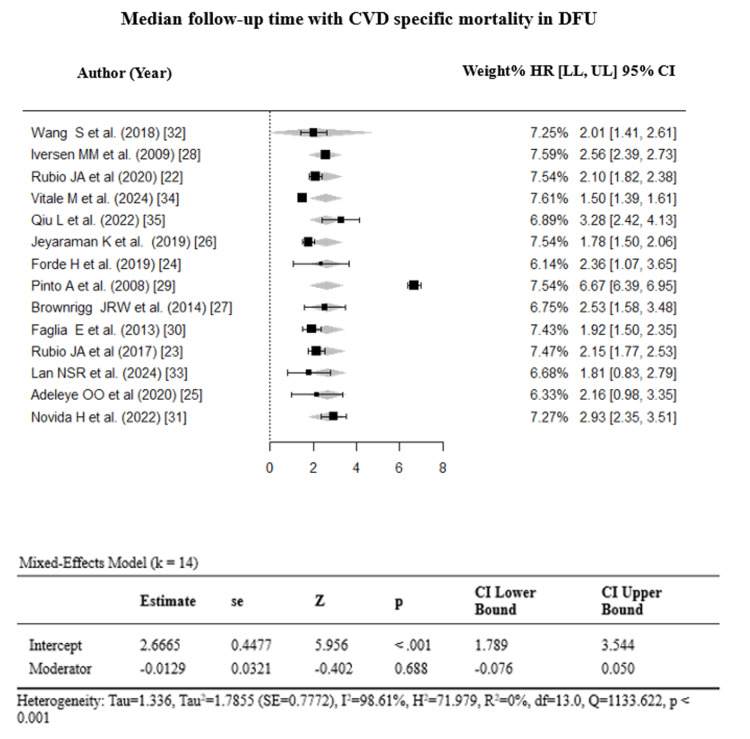
A forest plot to illustrate moderator analysis for median follow-up time. References: [22-35]. CVD: cardiovascular disease; DFU: diabetic foot ulcer; HR = hazard ratio; CI: confidence interval; LL: lower limit; UL: upper limit

**Figure 15:**
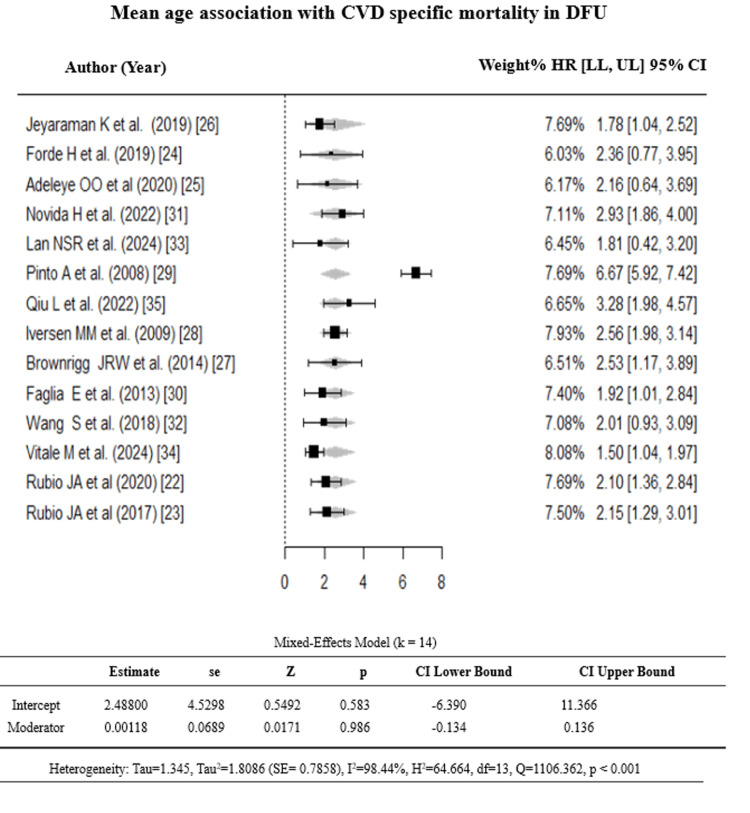
A forest plot to illustrate moderator analysis for mean age. References: [22-35]. CVD: cardiovascular disease; DFU: diabetic foot ulcer; HR = hazard ratio; CI: confidence interval; LL: lower limit; UL: upper limit

**Figure 16:**
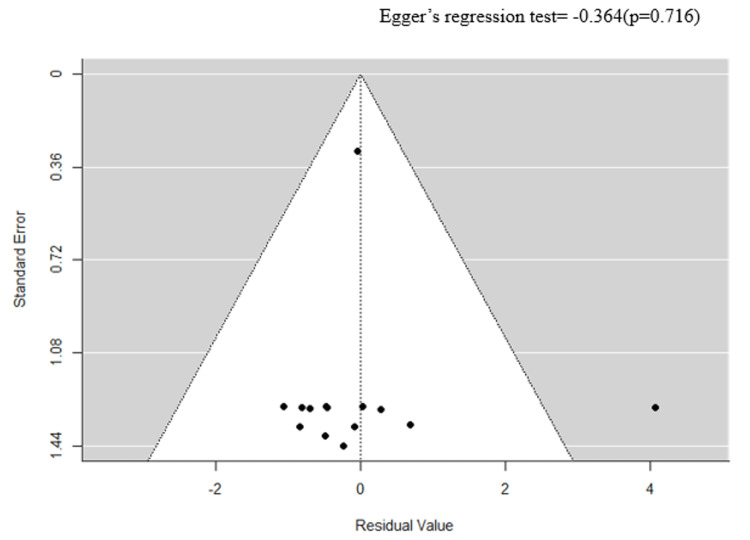
A funnel plot to illustrate moderator analysis for mean age.
